# Research on Alzheimer Disease in Italy: A Narrative Review of Pharmacological and Non-Pharmacological Interventions

**DOI:** 10.3390/neurolint17120196

**Published:** 2025-12-02

**Authors:** Miriana Caporlingua, Jole Castellano, Angelo Quartarone, Rosella Ciurleo

**Affiliations:** IRCCS Centro Neurolesi “Bonino-Pulejo”, Via Palermo S.S. 113, Contrada Casazza, 98124 Messina, Italy; miriana.caporlingua@irccsme.it (M.C.); jole.castellano@irccsme.it (J.C.); angelo.quartarone@irccsme.it (A.Q.)

**Keywords:** Alzheimer’s disease, Italy, preclinical studies, pharmacological treatments, non-pharmacological treatments, non-invasive neuromodulation techniques, multidisciplinary interventions

## Abstract

Background: Alzheimer’s Disease (AD) is the most common form of dementia and is characterized by progressive cognitive decline and neurodegeneration. In Italy, AD represents a major public health and socio-economic challenge. This review aims to summarize current Italian research on pharmacological and non-pharmacological interventions, including preclinical studies, clinical trials, rehabilitative approaches, and emerging neuromodulation techniques, highlighting contributions and future directions. Methods: A narrative review of the literature was conducted, focusing on Italian preclinical and clinical studies, observational and real-world evidence, cognitive and physical interventions, music therapy, non-invasive brain stimulation (rTMS, tDCS, tACS), and digital or home-based rehabilitation programs. Results: Italian research has explored different pharmacological strategies, including multitarget compounds, eptastigmine, rotigotine, and combinatorial therapies (donepezil-memantine, citicoline addition). Non-pharmacological interventions, such as cognitive stimulation, motor rehabilitation, music therapy, and multidimensional programs, demonstrated benefits on cognition, behavior, daily functioning, and caregiver well-being. Non-invasive neuromodulation techniques, targeting the dorsolateral prefrontal cortex and precuneus, showed promising effects on memory, attention, and executive functions, especially when combined with cognitive training. Digital health technologies, including telerehabilitation and home-based brain stimulation programs, further enhanced accessibility and adherence. Challenges remain due to fragmented research, small sample sizes, and limited standardization. Conclusions: Italian research on AD reflects a growing emphasis on integrated, multidimensional, and technologically advanced approaches. Strengthening preclinical studies, promoting multicenter collaborations, and combining pharmacological, cognitive, and neuromodulatory strategies may enhance therapeutic efficacy and patient quality of life. Continued investment in innovation and multidisciplinary research positions Italy to contribute meaningfully to global AD management and prevention.

## 1. Introduction

Alzheimer’s Disease (AD) is the most common form of dementia and represents a progressive and complex neurodegenerative disorder, which is characterized by the accumulation of neuritic plaques and neurofibrillary tangles resulting from the deposition of beta-amyloid peptide (Aβ) and phospho-tau. The most affected brain regions are the medial temporal lobe and neocortical structures [1,2,3].

Clinically, AD manifests as a progressive cognitive decline, including memory loss, impairment of language, learning abilities, visuospatial skills, reasoning, and behavior, strongly interfering with daily life and activities [4,5,6]. Advanced age represents the main risk factor, and in an increasingly aging society, the social and healthcare impact of the disease is expected to grow [7]. In this context, comorbidities are common in patients with AD and can further complicate disease management. Chronic conditions such as diabetes, cardiovascular diseases, depression, and inflammatory bowel diseases may precede or accompany AD progression [8,9,10,11]. The presence of comorbidities can negatively affect the efficacy of pharmacological and non-pharmacological treatments, accelerate disease progression, and increase overall clinical complexity. Accurate assessment of comorbidities is therefore essential to tailor therapeutic strategies and optimize patient care [12].

Despite advances in research, no drugs currently exist that can halt or reverse disease progression. Available therapies are symptomatic, aimed at slowing cognitive and behavioral decline. The main approved pharmacological classes include cholinesterase inhibitors (AChEIs), including donepezil, galantamine, and rivastigmine, and N-methyl-D-aspartate (NMDA) receptor antagonists, such as memantine. AChEIs increase acetylcholine levels by inhibiting acetylcholinesterase (AChE) and butyrylcholinesterase (BuChE) enzymes, while memantine counteracts NMDA receptor overactivation, preventing excessive Ca^2+^ influx and neurotoxicity [13,14,15,16,17,18,19,20]. These drugs allow for temporary symptom improvement but do not alter the biological course of the disease.

For this reason, disease-modifying therapies (DMTs), such as immunotherapies and small molecules, are under development to slow or halt AD pathophysiological processes, distinguishing themselves from purely symptomatic treatments [21,22,23].

Alongside pharmacological approaches, non-pharmacological interventions, including cognitive stimulation programs, rehabilitation, and digital technologies, are gaining increasing relevance in improving patients’ quality of life and reducing caregiver burden [24,25].

Globally, AD accounts for 60–70% of dementia cases. Currently, over 57 million people live with some form of dementia, with projections indicating up to 150 million cases by 2050 and approximately 10 million new diagnoses per year. Dementias are the seventh leading cause of death and have an economic impact exceeding $1 trillion annually, which is why the World Health Organization (WHO) and Alzheimer’s Disease International (ADI) have recognized them as a public health priority [26,27].

In Italy, it is estimated that approximately 1.2 million people live with some form of dementia, 50–60% of whom have AD (around 600,000 individuals). Projections indicate over 2.3 million cases by 2050, with a current prevalence of 5% among those over 60 years old [28,29]. Italy, one of the longest-living countries in the world, had 58.9 million inhabitants as of 1 January 2025, with 24.7% over 65, a percentage expected to rise to 34.5% by 2050 [30]. According to estimates from the Dementia Observatory of the Italian National Institute of Health, AD also imposes a substantial socio-economic burden, involving over 4 million people in daily care and an estimated cost of around €23 billion per year in direct and indirect expenses [31,32].

In light of these epidemiological data and the social impact of AD in Italy, it is crucial to analyze the contribution of national research to the development of new knowledge and therapeutic strategies.

This narrative review was conducted through a comprehensive search of the PubMed database, without applying temporal restrictions or predefined inclusion/exclusion criteria. The selection focused on studies addressing pharmacological and non-pharmacological interventions for AD conducted by Italian research groups. The aim of this review is to summarize the main evidence produced by these groups, with particular attention to both pharmacological and non-pharmacological approaches. Specifically, it will discuss preclinical and clinical study results on new molecules and pharmacological therapies, evidence related to rehabilitative and cognitive interventions, the emerging role of non-invasive neuromodulation (repetitive transcranial magnetic stimulation (rTMS), transcranial direct current stimulation (tDCS), and transcranial alternating current stimulation (tACS)), and the use of digital technologies and tele-rehabilitation, including updates on ongoing clinical studies. Despite the profound impact of AD and the limitations of current treatments, which are unable to halt progression or provide satisfactory therapeutic effects, this review provides an updated overview of Italian evidence, highlighting potential integrations among different approaches and outlining future perspectives and focusing on the role Italian research may play in the international AD landscape.

## 2. Pharmacological Treatments for Alzheimer Disease

Italian research on pharmacological treatments for AD encompasses a variety of innovative approaches, ranging from the synthesis and development of new multitarget molecules capable of acting on multiple pathogenic mechanisms to the analysis of data derived from observational studies and clinical trials.

### 2.1. Innovative Molecules and Multi-Target Strategies: Preclinical Studies

Preclinical research represents a crucial step in understanding the pathogenic mechanisms of AD and developing new therapeutic strategies. However, the Italian scientific output in this field remains limited and, to date, only a small number of preclinical studies have been published [33,34,35].

#### 2.1.1. In Vitro Studies

Montanari et al. [33] developed coumarin-based derivatives as an evolution of the AChEI 3-{4-[(benzylmethylamino)methyl]phenyl}-6,7-dimethoxy-2H-2-chromenone (AP2238), which is characterized by dual inhibitory activity on AChE and BuChE, an absence of neurotoxicity, and potential effectiveness in the early and intermediate stages of the disease. Some of these derivatives also demonstrated the ability to reduce Aβ42 aggregation and exert a neuroprotective effect against its toxicity. In this context, Carocci et al. [34] designed phenothiazine/donepezil-like hybrids with multitarget and antioxidant activity, including inhibition of AChE and BuChE, reduction of Aβ1-40 aggregation, and inhibition of the novel fatty acid amide hydrolase (FAAH), outlining a potential multifactorial therapeutic approach.

#### 2.1.2. In Vivo Studies

Mudò et al. [35] evaluated the effects of interferon-β1a (IFNβ1a) in a murine model of AD induced by Aβ1-42 peptide, observing an improvement in cognitive functions, a reduction in neuroinflammation with decreased levels of Interleukin-6 (IL-6) and Interleukin-1β (IL-1β) and increased Interleukin-10 (IL-10) and oxidative stress, alongside restoration of redox balance. Overall, IFNβ1a demonstrated neuroprotective potential, suggesting that strategies aimed at modulating neuroinflammation and oxidative stress may contribute to slowing disease progression.

### 2.2. Clinical Studies

Clinical studies represent a crucial step for the validation of new pharmacological therapies in AD, providing evidence on treatment efficacy, safety, and applicability in patient populations. Italian research has contributed to this field, exploring both next-generation cholinergic drugs and alternative therapeutic approaches, such as dopaminergic agonists and immunomodulators.

Among the first relevant studies, Imbimbo et al. [36] conducted a randomized, double-blind, placebo-controlled clinical trial in 349 patients with mild-to-moderate AD to assess the efficacy and safety of eptastigmine, an AChEI. Participants were treated for 25 weeks with eptastigmine at doses of 10 or 12 mg three times daily or with placebo. Treatment produced statistically significant improvements compared with placebo on the ADAS-Cog (AD Assessment Cognitive Subscale) (*p* = 0.047), CDR-SB (Clinical Dementia Rating Scale-Sum of the Boxes) (*p* = 0.010), and MMSE (Mini-Mental State Examination) (*p* < 0.001), with completion rates of 87% for eptastigmine and 82% for placebo. The drug was well tolerated, with mild cholinergic adverse events and treatment discontinuation in 5% of eptastigmine-treated patients versus 3% in the placebo group. These results demonstrate a positive effect of eptastigmine on cognitive function and overall performance in patients with mild-to-moderate AD.

Subsequent studies focused on dopaminergic agonists: Martorana et al. [37] observed, in a study of 17 AD patients, that rotigotine, a D_2_/D_3_ dopaminergic agonist administered transdermally in two separate sessions (4 mg/24 h for 4 days in Experiment 1; 2–6 mg/24 h for 4 days each in Experiment 2), increased cortical excitability and enhanced cholinergic transmission. These effects were evidenced by a reduction in short-interval intracortical inhibition (SICI), an increase in intracortical facilitation (ICF), and a potentiation of short-latency afferent inhibition (SLAI). Such changes were not observed in the 8 healthy controls (HCs), were dose-dependent, and were not associated with adverse events. These findings support an early involvement of the dopaminergic system in the pathogenesis of AD and suggest a potential therapeutic role for rotigotine in modulating cortical circuits and cholinergic function.

Koch et al. investigated the role of rotigotine in AD in two separate studies. In the first, published in 2014, they assessed the effect of rotigotine on cortical plasticity in 30 patients with mild AD, divided into three groups treated with rotigotine, rivastigmine, or placebo, compared with HCs before and after 4 weeks of treatment. Cortical plasticity was evaluated via rTMS using theta burst stimulation (TBS) protocols, and cholinergic activity using a short-latency afferent inhibition (SAI) protocol. At baseline, patients with AD exhibited reduced long-term potentiation (LTP)-like plasticity; rotigotine restored this plasticity and enhanced cholinergic activity, effects that persisted up to 12 weeks. Rivastigmine only increased cholinergic activity, while placebo had no effect. Cognitively, rotigotine improved MMSE and Frontal Assessment Battery (FAB) scores, with effects maintained at 12 weeks, whereas rivastigmine only increased MMSE scores and placebo showed no significant changes. The treatment was well tolerated and free of adverse events. These findings indicate that rotigotine can effectively modulate cortical plasticity and cholinergic activity, suggesting a potential therapeutic role for dopaminergic agonists in cortical circuits and cognitive function in patients with mild AD [38].

The second study, published in 2020, was a phase IIa, randomized, double-blind, placebo-controlled clinical trial involving 94 patients with mild-to-moderate AD treated for 24 weeks. Participants were divided into two groups: transdermal rotigotine (4 mg/24 h) in addition to rivastigmine (AChEI) or placebo plus standard therapy. Rotigotine did not result in a significant improvement in global cognitive function, with an estimated mean change in ADAS-Cog score of 2.92 in the rotigotine group compared to 2.66 in the placebo group. However, secondary outcomes showed clinically relevant benefits: the decline in activities of daily living, measured with ADCS-ADL, was more moderate in the rotigotine group (−3.32) compared to placebo (−7.24), and executive functions assessed with the FAS improved in the rotigotine group (+0.48) while they worsened in the placebo group (−0.66). Neurophysiological analysis also highlighted an increase in prefrontal cortical activity in the rotigotine group, but not in the placebo group, suggesting a targeted effect on frontal circuits. Overall, these results confirm that, although rotigotine did not improve global cognition, it can enhance frontal executive functions and maintain autonomy in daily activities, suggesting the utility of a combined cholinergic-dopaminergic therapeutic approach in the early stages of the disease [39]. Finally, Grimaldi et al. [40] conducted a phase II, multicenter, randomized, double-blind, placebo-controlled trial in 42 patients with mild AD treated for 28 weeks with subcutaneous IFNβ1a (three times per week) or placebo, followed by 24 weeks of follow-up. The treatment was safe and well tolerated, without statistically significant differences in primary cognitive outcomes, but showed a slowing of cognitive decline during follow-up and significant improvement in daily activities. At baseline, neuropsychological test scores did not differ significantly between the two groups: the ADAS-Cog score was 19.81 ± 6.14 in the placebo group and 18.34 ± 7.85 in the IFNβ1a group (*p* = 0.34), while the MMSE score was 22.93 ± 1.78 vs. 23.45 ± 2.13 (*p* = 0.41). During the 28 weeks of treatment, there were no statistically significant differences in primary cognitive outcomes (ADAS-Cog) between IFNβ1a and placebo. However, during the post-treatment follow-up, the treated group showed a slower rate of cognitive decline compared to placebo. Furthermore, during the follow-up, the IFN-β1a-treated group showed significant improvements in daily living: IADLs increased by 0.94 ± 0.37 (*p* = 0.0306) after treatment and by 1.20 ± 0.59 (*p* = 0.041) at follow-up compared to the placebo group. Overall, these results indicate that, despite not showing significant cognitive improvements in the primary tests, IFNβ1a appears to modulate functional decline in activities of daily living, suggesting a potential protective effect in the early stages of the disease, strengthening the hypothesis of its possible therapeutic role in AD, worthy of further, larger-scale studies.

Although these studies provide valuable insights into cholinergic, dopaminergic, and immunomodulatory pathways, their implications for clinical practice remain limited. Most interventions showed mechanistic or domain-specific benefits (e.g., plasticity, executive functions, daily activities), but none produced clinically meaningful changes in global cognition or demonstrated sufficient evidence to modify current treatment guidelines. These findings refine our understanding of early therapeutic targets and suggest potential future combination strategies, yet their translation into routine care requires larger, multicenter trials with functional and long-term outcomes.

The main Italian clinical studies on pharmacological interventions in Alzheimer’s disease are summarized in Table 1.

### 2.3. Pharmacological Treatments in Clinical Practice: Observational and Real-World Evidence

Baldereschi et al. [41] showed an association between estrogen replacement therapy and a reduced risk of AD in postmenopausal women, suggesting a potential protective effect against cognitive decline. Subsequently, Castagna et al. [42] highlighted that the addition of citicoline to standard therapy with memantine and AChEIs improves the maintenance of cognitive functions, although without differences in functional autonomy or behavioral symptoms. More recently, Padovani et al. [43] described, through real-world data, the widespread use of the extemporaneous combination of donepezil and memantine in Italy, underlining the utility of a fixed-dose formulation to improve therapeutic adherence and patient management, reducing the healthcare burden.

## 3. Non-Pharmacological Interventions in Alzheimer’s Disease

Alongside pharmacological treatments, in recent years, there has been growing interest in non-pharmacological approaches aimed at supporting cognitive function, reducing behavioral symptoms, and improving the quality of life of patients with AD. These include cognitive training (CT) and cognitive stimulation programs, physical and environmental interventions adapted to the different stages of the disease, and integrated strategies combined with pharmacological treatments. Collectively, these interventions aim to preserve residual abilities, slow functional decline, and address the complexity of the disease from a multidimensional perspective.

### 3.1. Cognitive Training and Cognitive Stimulation

CT and stimulation aim to support residual functions and contain decline in the early stages of AD, with positive effects on both cognitive abilities and daily behavior of patients.

Farina et al. explored various CT approaches in patients with mild-to-moderate AD. In a pilot study involving 22 patients, they compared individual training based on procedural memory stimulation with a program targeting residual cognitive functions. Both groups showed improvements in daily living skills, with additional benefits in attention and verbal fluency for the procedural memory group, although these effects tended to diminish after three months [44].

Subsequently, in a larger group study involving 32 patients, the authors compared a “global” stimulation approach (recreational activities integrated with psychological support) with a “cognitive-specific” approach focused on procedural memory exercises and rehabilitation of residual functions. In this case, global stimulation significantly improved behavioral symptoms, daily functional abilities, and verbal fluency, while also reducing caregiver burden, whereas the cognitive-specific approach produced more limited benefits, mainly restricted to functional abilities [45].

Later, Baglio et al. [46] evaluated the efficacy of Multidimensional Stimulation Group Therapy (MST) in 60 patients with mild-to-moderate AD, randomly assigned to MST (tMST) or standard care without stimulation (ntMST) for 10 weeks, with a follow-up at 22 weeks for the tMST group. MST demonstrated a significant reduction in behavioral and psychological symptoms of dementia (BPSD), improvements in memory and language abilities, and increased brain activity in temporal areas, right insular cortex, and thalamus. Overall, these findings support the effectiveness of MST in enhancing the cognitive-behavioral status of patients, promoting recovery of residual functions, motivation, and the prevention of further decline.

### 3.2. Physical Interventions

Physical strategies aim to stimulate motor and cognitive functions through simple, adapted activities, such as walking or structured games. In particular, Venturelli et al. [47] evaluated a six-month walking program in 21 patients with advanced AD, randomly assigned to a Walking Group (WG, *n* = 11) or Control Group (CG, *n* = 10), and observed improvements in motor function, daily activities, and a slowing of cognitive decline compared to the CG. The following year, the same group studied the acute effects of adapted games (AG) in 20 patients with advanced dementia. Participants underwent a single 30-min session of AG or placebo activities. The results showed a reduction in agitation and an increase in cognitive performance, suggesting a potential role of dopaminergic and serotonergic stimulation and greater activation of the prefrontal cortex [48].

### 3.3. Combined Therapies

Combined therapies integrate non-pharmacological interventions with pharmacological or rehabilitative treatments. In this context, Giovagnoli et al. [49] evaluated, in a randomized trial, the effect of active music therapy (AMT) added to memantine in 45 patients with moderate AD already receiving AChEI therapy. The 24-week treatment did not improve language but reduced symptoms of depression and appetite disturbances, mitigating psycho-behavioral deterioration.

Fonte et al. [50] conducted a six-month randomized study on 87 patients with Mild Cognitive Impairment (MCI) or AD, comparing CT, physiotherapy (PT), and CG. Both CT and PT slowed cognitive decline compared to the control, maintaining stable Mini-Mental State Examination (MMSE) scores, with memory improvements observed in MCI; PT additionally benefited cardiovascular factors. These results support the combined use of CT and PT as effective non-pharmacological strategies for MCI and AD, highlighting the need for continuous interventions, particularly in AD.

The Italian evidence on non-pharmacological interventions indicates a stronger and more immediate translatability to clinical practice compared with emerging pharmacological treatments. Cognitive training, multidimensional stimulation, and structured physical activity consistently improve daily functioning, behavioral symptoms, and caregiver burden, making them readily implementable in clinical and community settings. Music therapy and combined CT–physiotherapy approaches show additional benefits on mood, behavior, and cardiovascular health, reinforcing the value of integrated, continuous interventions.

A summary of the main Italian clinical studies investigating non-pharmacological and combined interventions in AD is reported in Table 2.

## 4. Non-Invasive Neuromodulation Techniques

The use of brain neurostimulation through TMS, rTMS, and anodal or cathodal tDCS and tACS has been attracting increasing interest in the scientific community. These non-invasive techniques offer the possibility to modulate brain activity in a targeted manner, allowing the exploration of new brain functions and the development of potential therapeutic strategies [51,52,53].

### 4.1. Transcranial Magnetic Stimulation/Repetitive Transcranial Magnetic Stimulation

TMS and rTMS are indirect, non-invasive techniques that allow for the modulation of motor cortex excitability through pulses generated by a coil producing a magnetic field capable of penetrating the scalp. TMS is primarily used to study brain function, whereas rTMS is employed to induce changes in cortical activity that can persist even after stimulation ends [54]. The magnitude and duration of these effects depend on the stimulation frequency: low frequencies (≤1 Hz) tend to reduce cortical excitability, while high frequencies (≥5 Hz) increase it [55]. Over the past two decades, numerous clinical studies have demonstrated that rTMS can improve AD symptoms, confirming its potential as a therapeutic approach [56].

Beyond its therapeutic use, TMS can provide valuable information on brain function and connectivity, suggesting a possible role as a diagnostic tool. In this context, Benussi et al. [57] showed that intracortical connectivity measures obtained via TMS can enhance diagnostic accuracy in AD, with performance comparable to amyloidosis biomarkers, highlighting their potential as an additional screening marker alongside standard clinical tools.

#### 4.1.1. rTMS on the Dorsolateral Prefrontal Cortex

The dorsolateral prefrontal cortex (DLPFC) is one of the main target regions in rTMS studies for the treatment of AD, given its involvement in higher cognitive processes such as memory, attention, and executive functions [58]. Several studies have investigated the efficacy of rTMS on this area, showing promising results with both high-frequency excitatory protocols and low-frequency inhibitory protocols, depending on the stimulated hemisphere and the targeted cognitive function. Cotelli et al. [59] demonstrated that 4 weeks of high-frequency rTMS over the DLPFC improved language comprehension in 10 patients with AD, with effects lasting up to 8 weeks.

Turriziani et al. [60] investigated low-frequency (1 Hz) inhibitory rTMS over the right DLPFC in mild AD. In the first experiment (24 patients), right DLPFC stimulation improved recognition memory, while left DLPFC had no effect. In the second experiment (14 patients), repeated two-week right DLPFC rTMS led to improvements persisting at one-month follow-up. The results suggest that right DLPFC rTMS may represent an effective non-pharmacological therapeutic option for the treatment of specific cognitive deficits in AD.

#### 4.1.2. rTMS on the Precuneus

New therapeutic targets, such as the precuneus (PC), may represent more effective intervention sites for improving memory in patients with AD [61]. In this context, several studies have explored the efficacy of rTMS targeted to the PC, aiming to modulate cortical activity and memory-related functional connectivity.

Koch et al. [62] evaluated, in a two-week randomized, crossover, double-blind, placebo-controlled study, the effect of high-frequency rTMS over the PC in 14 patients with early-stage AD. Stimulation improved episodic memory and, according to electroencephalography (EEG), increased neural activity, enhanced beta oscillations, and improved functional connectivity with the medial frontal areas of the default mode network (DMN). In subsequent trials of 24 weeks [63] and 52 weeks [64], PC rTMS slowed cognitive and functional decline, improved daily autonomy, and reduced behavioral disturbances, demonstrating safety and good tolerability.

#### 4.1.3. rTMS Combined with Cognitive Training

Two Italian clinical studies have explored the efficacy of rTMS as a complement to CT in patients with mild-to-moderate AD. Bagattini et al. [65] conducted a multicenter, randomized, double-blind, sham-controlled trial on 50 patients, applying high-frequency rTMS over the left DLPFC combined with associative face-name memory training for 4 weeks. The group receiving real rTMS showed significant improvements in associative memory and in untrained cognitive functions, maintained for at least 12 weeks, with stronger effects in patients with less severe impairment and higher educational levels.

Vecchio et al. [66] conducted a randomized, double-blind, sham-controlled trial on 72 patients with mild-to-moderate AD, combining neuronavigated rTMS over six specific brain regions identified by Magnetic Resonance Imaging (MRI) with CT for six weeks, with a 40-week follow-up. The treatment produced immediate cognitive improvements and, in the long term, modulation of brain connectivity was observed via EEG, with delta and alpha1 bands indicative of diagnostic value and alpha2 predictive of cognitive recovery.

Both studies highlight the potential of rTMS, both standard and neuronavigated, as a non-pharmacological strategy to enhance CT in AD, producing effects on cognitive performance as well as brain network modulation.

#### 4.1.4. rTMS Using H-Coil

Among the various rTMS configurations, the use of the H-coil stands out for its ability to penetrate deeper and more broadly into brain areas compared to traditional coils. This allows for the modulation of more extensive neural circuits potentially involved in cognitive processes impaired in AD, thereby increasing the chances of therapeutic efficacy. The H-coil, due to its particular shape and technology, can simultaneously stimulate multiple bilateral cortical regions, offering a more global approach to neuromodulation [67].

In this context, Leocani et al. [68] conducted a randomized, double-blind, placebo-controlled trial in 30 patients with AD, evaluating rTMS with the H2-coil at 10 Hz over bilateral fronto-temporo-parietal areas for 4 weeks, followed by 4 weeks of maintenance. Real stimulation produced a transient improvement in Alzheimer’s Disease Assessment Scale-cognitive (ADAS-Cog) scores without effects on MMSE scores or the Beck Depression Inventory-II (BDI-II). The study confirms that H-coil rTMS is safe, feasible, and potentially useful as a non-pharmacological intervention, suggesting further research in early stages of the disease and in combination with cognitive rehabilitation.

### 4.2. Transcranial Alternating Current Stimulation

tACS is a non-invasive neuromodulation technique (NIBS) that modulates cortical oscillations by aligning them (entrainment) with the frequency of the applied current, and has been shown to alter oscillatory activity, improving cognitive abilities in healthy adults. This technique represents a safe and painless approach to modulate cortical excitability and influence neuroplasticity, with the ultimate goal of enhancing cognitive and behavioral processes [69].

In this context, Benussi et al. conducted two randomized, double-blind, placebo-controlled, crossover pilot studies to evaluate the efficacy of gamma-frequency-tACS (γ-tACS) over the PC/medial parietal cortex in patients with MCI-AD or early AD. The first study [70] was conducted on 20 patients and the second [71] was conducted on 60 patients in which a single 60-min session of real γ-tACS was found to significantly improved episodic memory and long-term recall, restoring cholinergic transmission in association with increased gamma activity in posterior regions. Efficacy was greater in patients with mild-stage disease and in non-carriers of the ApoE ε4 allele. The stimulation was safe and promising as a non-invasive strategy, with potential for multi-session and home-based applications.

### 4.3. Transcranial Direct Current Stimulation

tDCS is a non-invasive technique capable of inducing lasting functional changes in brain activity. Current evidence shows that this simple and safe procedure can modulate numerous brain functions, both motor and cognitive, in clinical settings, including motor cortical inhibition, gait adaptation, learning, language, memory and attention [72]. Recent studies have explored the therapeutic potential of tDCS in AD, with a particular focus on improving cognitive functions.

Ferrucci et al. [73] investigated the cognitive effects of tDCS over the temporoparietal areas in 10 patients with AD. Anodal stimulation improved word recognition memory task and cathodal stimulation worsened it while sham stimulation had no effect.

Boggio et al. [74] studied the efficacy of anodal tDCS applied to the temporal cortex in 15 patients with AD, which was administered in five consecutive daily stimulation sessions. The stimulation produced a significant improvement in visual recognition memory, which persisted for at least 4 weeks after the end of treatment.

Cotelli et al. [75] conducted a randomized, double-blind pilot study on 36 patients with AD to evaluate the effect of anodal tDCS combined with personalized mnemonic training on face-name associations. Participants received real or sham tDCS paired with mnemonic or motor training for two weeks. The results showed a significant improvement in face-name association performance in all patients undergoing mnemonic training, with effects maintained for up to three months while tDCS alone did not provide additional benefits.

Fonte et al. [76] conducted a randomized, double-blind, sham-controlled pilot study on 23 patients with mild-to-moderate AD to evaluate the efficacy of anodal tDCS combined with motor or cognitive activity. Participants received 15 min of stimulation over the DLPFC during 45-min activity sessions, five days per week for two weeks. Real tDCS significantly improved global cognitive status and attention compared to sham, with a more pronounced effect when combined with motor activity at one-week follow-up.

Overall, these findings indicate that anodal tDCS can improve memory and cognitive functions in patients with AD, with greater benefits when combined with CT or motor activity. The results support further research on repeated protocols and combined interventions, aiming to enhance cognitive functions and slow disease progression.

The clinical evidence from studies on rTMS, tDCS, and tACS suggests that non-invasive neuromodulation can provide meaningful cognitive benefits in patients with AD, particularly in memory, attention, and language functions. rTMS targeting the DLPFC or precuneus has been shown to improve cognitive performance and slow functional decline, indicating potential use in early-to-moderate stages of AD. Combining neuromodulation with cognitive or motor training enhances and prolongs these effects, highlighting the value of integrated rehabilitation programs. Overall, these interventions are safe and well-tolerated, supporting their inclusion in specialized memory clinics or research-oriented clinical programs.

The main clinical studies investigating non-invasive brain stimulation techniques in patients with AD are summarized in Table 3.

## 5. From Early Diagnosis to Neuromodulation: Ongoing and Planned Research in Italy

Italian research on AD is rapidly evolving, driven by the development of increasingly sophisticated and multidisciplinary therapeutic strategies. Alongside traditional pharmacological approaches, new strategies are emerging that range from synaptic modulation to combined therapies for the treatment of cognitive and behavioral symptoms.

At the preclinical level, the ENERGISE study [77] aims to modulate synaptic plasticity through the use of engineered proteins, while in the clinical setting, a Phase III trial [78] is currently ongoing to evaluate the efficacy of a combined therapy with rotigotine and rivastigmine.

On the diagnostic front, high-tech tools are emerging, such as the Fundus Camera Module [79], which uses multispectral fundus imaging to detect early alterations associated with AD. At the same time, rehabilitative interventions are enriched with innovative modalities, including virtual reality and telemedicine-based programs [80], designed to make cognitive rehabilitation accessible directly at home.

Other studies have tested complementary approaches: the ABCD study [81] evaluated Environmental Ecological Therapy to reduce behavioral disturbances in patients with advanced Alzheimer’s while another study [82] examined the efficacy of cognitive stimulation in patients with mild disease or mild cognitive impairment.

Special attention is given to non-invasive neuromodulation, with protocols using rTMS, tACS, and tDCS to improve cognitive functions and slow cognitive decline. Among the most innovative interventions are personalized home-based programs, such as NEUROTWIN [83] and tACS@Home [84], as well as studies aimed at enhancing autobiographical memory and/or brain connectivity, including pADmemory [85], StimoLaMente [86], and Network-based rTMS in AD [87].

The protocols described by Pievani et al. [88] and Altomare et al. [89] exemplify advanced study designs, combining neuropsychological, neurophysiological, and neuroimaging assessments to monitor the effects of brain stimulation.

Overall, these trials reflect the growing commitment of Italian research to develop increasingly innovative strategies to prevent, slow, or modulate the progression of AD, offering promising prospects for future therapeutic interventions and preventive approaches.

## 6. Discussion and Conclusions

In this review, we described the progress of Italian research on AD, highlighting how, in recent years, various areas of intervention have been explored, ranging from pharmacological approaches to rehabilitation and non-pharmacological strategies. This variety reflects the growing awareness that Alzheimer’s cannot be managed with a single treatment, but requires an integrated and multidimensional approach.

In the preclinical field, Italian research is still less developed compared to other countries, but the available contributions have investigated key mechanisms such as neuroinflammation, oxidative stress, and cholinergic dysfunction. Some studies have reported encouraging results, for example, with IFNβ1a and multitarget compounds with cholinergic, antioxidant, and anti-amyloid properties. Strengthening this area will be essential to accelerate the translation of laboratory findings into clinical practice.

At the clinical level, Italian studies have contributed to the evaluation of new molecules and pharmacological approaches at different stages of clinical testing. Experimental studies have investigated agents such as rotigotine and eptastigmine, while observational and real-world studies have assessed combinatorial strategies, including donepezil-memantine or the addition of citicoline, suggesting potential synergies between different neurotransmitter systems.

Compared to pharmacological approaches, non-pharmacological interventions have shown even broader development and represent a strong point of Italian research. Cognitive stimulation techniques, motor rehabilitation, music therapy, and multidimensional approaches such as MST have demonstrated positive effects not only on cognitive functions but also on behavior, daily activities, and caregiver well-being.

Another rapidly expanding field is non-invasive brain stimulation (rTMS, tDCS, tACS), which has shown good tolerability and promising results in improving memory, attention, and executive functions. In particular, targeted stimulation of regions such as the precuneus and dorsolateral prefrontal cortex introduces new perspectives for early interventions. At the same time, the growing adoption of digital health technologies, such as virtual reality, telerehabilitation, and home-based brain stimulation programs (e.g., NEUROTWIN, tACS@Home), represents a distinctive feature of Italian research, improving accessibility, adherence, and sustainability of care.

Despite the valuable contributions of Italian research, some limitations should be highlighted. Many studies had small sample sizes, which may impact the robustness and generalizability of the findings [39,47,48,50,53,60,62,70,71,74,76]. Non-pharmacological interventions were often implemented with heterogeneous protocols in terms of duration, frequency, and outcome measures, making direct comparisons difficult. Furthermore, follow-up periods were sometimes short, limiting the evaluation of long-term effects [46,53,65,75,76]. Highlighting these limitations is essential to correctly interpret the results and design more robust future studies, including the expansion of cohorts and the adoption of shared protocols to ensure greater reliability and impact. Although Italy is highly active in collaborative research networks and in the development of innovative non-pharmacological and digital approaches, the country lacks a systematic comparison between its Alzheimer’s research program and those of other countries [90]. Moreover, a major structural barrier concerns the overall deficiency of national research funding, which remains significantly lower than in many European and non-European settings. This underfunding can restrict the possibility to expand cohorts, consolidate multicenter projects, and conduct long-term studies. Strengthening multicenter collaborations, adopting shared methodologies, and developing comparative assessments of national research programs would therefore be fundamental to aligning Italy with the most competitive European research contexts and fully leveraging its potential in international dementia research. Italy’s position must also be considered within a broader international landscape. In Ibero-America, research has addressed tau hyperphosphorylation and self-assembly, beta-amyloid, microglial dysfunction and neuroinflammation, alongside clinical trials on immunotherapies [91]. An overview of the main genetic variants associated with AD and frontotemporal dementia in Latin American populations highlights the region’s marked genetic heterogeneity and the predominant role of mutations such as PSEN1, GRN, and MAPT [92]. Major barriers to Alzheimer’s diagnosis and treatment have also been identified across several Latin American countries, with practical recommendations proposed to improve clinical training, diagnostic tools, access to care, and region-specific genetic strategies [93]. Additionally, a study conducted in Mexico emphasized the multifactorial nature of AD in the region, identifying key risk factors such as age, genetics, and metabolic comorbidities, and reported the advances in diagnostic techniques, particularly for early biomarkers [94]. Japan has investigated the relationship between amyloid plaques and tau tangles, as well as the role of presenilins and ApoE polymorphisms [95]. Australia has distinguished itself in the development of advanced biomarkers, anti-tau immunotherapies and microglia studies, as well as research on vascular factors and the gut–brain axis [96]. Finally, China has confirmed the efficacy of standard drugs such as rivastigmine and donepezil for mild-to-moderate AD, and developed GV-971, a sodium oligomannate, which improves cognition by modulating the gut microbiota [97].

These international contributions ranging from molecular mechanisms to clinical interventions offer valuable insights that Italian research could integrate. In particular, Italy could further develop approaches combining pharmacological treatments with cognitive stimulation, neuromodulation, and digital tools while also exploring emerging pathways, such as the role of the gut microbiome in AD, in light of the growing evidence for the gut–brain axis in its pathogenesis, and develop advanced gene- or cell-based therapeutic strategies aimed at modifying genetic risk factors and enhancing neuroprotection [98,99,100,101].

These future directions could provide key insights for truly disease-modifying strategies and innovative interventions.

Finally, it should be noted that AD is a complex, multifactorial condition: adopting integrated therapeutic strategies capable of acting simultaneously on multiple pathogenic pathways may be key to achieving more robust and lasting results.

In conclusion, Italian research is moving toward a more innovative, personalized, and technologically advanced vision. By investing in innovation, enhancing multidisciplinary collaboration, and consolidating ongoing or planned projects, Italy could play an increasingly important role in the global fight against Alzheimer’s, contributing to new therapeutic strategies and improving the quality of life for patients and caregivers. In this context, the Italian government supports AD and other dementias primarily through the Alzheimer and Dementia Fund 2024–2026, aiming to ensure continuity of care, strengthen territorial services, and promote innovative and integrated interventions in line with the National Dementia Plan [102,103].

## Figures and Tables

**Table 1:** Summary of Italian clinical studies on pharmacological treatments in Alzheimer’s disease.

Study	Study Design	Drug	Population	Treatment Duration	Main Findings
Imbimbo et al. [36]	Randomized, double-blind, placebo-controlled clinical trial	Eptastigmine (AChEI)	349 patients with mild-to-moderate AD	25 weeks	Significant improvements were observed in cognition and global performance following treatment.
Martorana et al. [37]	Controlled Clinical Trial	Rotigotine (D_2_/D_3_ dopaminergic agonist)	17 AD patients; 8 HCs	4 days (Exp. 1); 4 days × 3 doses (Exp. 2)	Rotigotine increased cortical excitability and restored cholinergic transmission.
Koch et al. [38]	RCT	Rotigotine, rivastigmine, or placebo	30 patients with mild AD + HCs	4 weeks	Rotigotine normalized LTP-like plasticity, while rivastigmine and placebo had no impact; cholinergic activity was enhanced by both rotigotine and rivastigmine.
Koch et al. [39]	Phase IIa, randomized, double-blind, placebo-controlled clinical trial	Rotigotine + rivastigmine vs. placebo + standard therapy	94 patients with mild-to-moderate AD	24 weeks	Rotigotine had no impact on global cognitive functions but improved executive and frontal abilities as well as independence in daily activities.
Grimaldi et al. [40]	Phase II, multicenter, randomized, double-blind, placebo-controlled trial	Subcutaneous IFNβ1a (immunomodulator) or placebo	42 patients with mild AD	28 weeks + 24 weeks follow-up	No significant effect on primary cognitive outcomes; slowed cognitive decline during follow-up and enhanced daily function.

Legend: AChEI: Acetylcholinesterase Inhibitor; AD: Alzheimer’s Disease; D_2_/D_3_: Dopamine receptor subtypes 2 and 3; HCs: Healthy Controls; IFNβ1a: Interferon-beta-1a; LTP: long-term potentiation; RCT: Randomized Controlled Trial.

**Table 2:** Summary of Italian clinical studies on non-pharmacological interventions in Alzheimer’s disease.

Study	Study Design	Type of Intervention	Population	Duration	Main Findings
Farina et al. [44]	Pilot study	CT (procedural memory vs. residual cognitive functions)	22 patients with mild-to-moderate AD	5 weeks + 3 months follow-up	Both groups demonstrated progress in daily living abilities, while the procedural memory group additionally showed improvements in attention and verbal fluency; these effects decreased after 3 months.
Farina et al. [45]	Comparative clinical trial	Group CT: ‘global’ stimulation vs. ‘cognitive-specific’ approach	32 patients with mild-to-moderate AD	6 weeks + 6 months follow-up	Global stimulation improved behavioral symptoms, daily functioning, and verbal fluency, and reduced caregiver burden; cognitive-specific training showed limited benefits, mainly in functional abilities.
Baglio et al. [46]	RCT	MST	60 patients with mild-to-moderate AD	10 weeks + 22 weeks follow-up (tMST group only)	MST resulted in significant improvements in BPSD, memory and language abilities, and increased neural activity in temporal areas, right insular cortex, and thalamus.
Venturelli et al. [47]	RCT	Walking program	21 patients with advanced AD: 11 WG + 10 CG	6 months	Improvements in motor function, daily activities, and a slowing of cognitive decline.
Venturelli et al. [48]	RCT	AGs vs. Placebo	20 patients with advanced dementia	Single 30-min session	Lower levels of agitation and higher cognitive performance.
Giovagnoli et al. [49]	RCT	AMT + memantine	45 patients with moderate AD	24 weeks	No language improvement; reduced depression and appetite disturbances, mitigating psycho-behavioral deterioration.
Fonte et al. [50]	RCT	CT vs. PT	87 MCI or AD patients	6 months + 3 months follow-up	CT and PT slowed cognitive decline (stable MMSE scores); memory improved in MCI; PT improved cardiovascular factors.

Legend: AD: Alzheimer’s Disease; AGs: Adapted Games; AMT: Active Music Therapy; BPSD: Behavioral and Psychological Symptoms of Dementia; CG: Control Group; CT: Cognitive Training; MCI: Mild Cognitive Impairment; MMSE: Mini-Mental State Examination; MST: Multidimensional Stimulation Group Therapy; PT: Physiotherapy; RCT: Randomized Controlled Trial; tMST: Treated with Multidimensional Stimulation Group Therapy; WG: Walking Group.

**Table 3:** Summary of clinical studies on non-invasive neuromodulation techniques in Alzheimer’s disease.

Study	Technique	Population	Target Area	Duration	Main Findings
Cotelli et al. [59]	rTMS (high-frequency)	10 AD patients	DLPFC	4 weeks	Improved language comprehension, with effects lasting up to 8 weeks.
Turriziani et al. [60]	rTMS (1 Hz)	24 + 14 patients with mild AD	Left/right DLPFC	First experiment: single-session; Second experiment: 2 weeks + 1 month follow-up	Right DLPFC stimulation improved recognition memory; effects persisted at 1-month follow-up.
Koch et al. [62]	rTMS (high-frequency)	14 patients with early-stage AD	PC	2 weeks	Improved episodic memory, increased neural activity, enhanced beta oscillations, and strengthened connectivity with medial frontal DMN areas.
Koch et al. [63]	rTMS	50 patients with mild-to-moderate AD	PC	24 weeks	Slowed cognitive and functional decline, improved daily autonomy, and reduced behavioral disturbances.
Koch et al. [64]	rTMS	48 patients with mild-to-moderate AD	PC	52 weeks	Slowed cognitive and functional decline, improved daily autonomy, and reduced behavioral disturbances.
Bagattini et al. [65]	rTMS (high frequency) + CT	50 patients with mild-to-moderate AD	Left DLPFC	4 weeks + 12 weeks follow-up	Improved associative memory and untrained cognitive functions; effects lasted ≥ 12 weeks.
Vecchio et al. [66]	rTMS + CT	72 patients with mild-to-moderate AD	Six cortical regions (MRI-guided)	6 weeks + 40 weeks follow-up	Immediate cognitive improvements and long-term modulation of brain connectivity.
Leocani et al. [68]	rTMS using H2-coil (10 Hz)	30 AD patients	Bilateral fronto-temporo-parietal areas	4 weeks + 4 weeks with maintenance treatment	Transient improvement in ADAS-Cog scores; no effects on MMSE or BDI-II scores.
Benussi et al. [70]	γ-tACS	20 patients with MCI-AD	PC/medial parietal cortex	Single 60-min session	Significantly improved episodic memory and long-term recall, with restored cholinergic transmission.
Benussi et al. [71]	γ-tACS	60 patients with early AD	PC	Single 60-min session	Significantly improved episodic memory and long-term recall, with restored cholinergic transmission.
Ferrucci et al. [73]	tDCS (anodal vs. cathodal vs. sham)	10 patients with AD	Temporoparietal areas	3 separate sessions with ≥1-week interval	Anodal stimulation improved word recognition memory task and cathodal stimulation worsened it while sham stimulation had no effect.
Boggio et al. [74]	tDCS (anodal)	15 AD patients	Temporal cortex	5 days + 4 weeks follow-up	Significant improvement in visual recognition memory, which persisted for at least 4 weeks after the end of the treatment.
Cotelli et al. [75]	Anodal tDCS during face-name associations memory training	36 AD patients	Left DLPFC	2 weeks + 6 months follow-up	Significant improvement in face-name association performance in all patients, with effects lasting up to 3 months.
Fonte et al. [76]	Anodal tDCS + motor or cognitive activity.	23 patients with mild-to-moderate AD	DLPFC	2 weeks + 1 week follow-up	Global cognitive status and attention were significantly enhanced, with greater effects observed when combined with motor activity at the 1-week follow-up.

Legend: ADAS-Cog: Alzheimer’s Disease Assessment Scale Cognitive; AD: Alzheimer’s Disease; BDI II: Beck Depression Inventory Scale-II; CT: Cognitive Training; DLPFC: Dorsolateral Prefrontal Cortex; DMN: Default Mode Network; γ-tACS: Gamma-frequency Transcranial Alternating Current Stimulation; Hz: Hertz; MCI: Mild Cognitive Impairment; MMSE: Mini-Mental State Examination; MRI: Magnetic Resonance Imaging; PC: Precuneus; rTMS: Repetitive Transcranial Magnetic Stimulation; tACS: Transcranial Alternating Current Stimulation; tDCS: Transcranial Direct Current Stimulation.

## Data Availability

Not applicable.

## Abbreviations

The following abbreviations are used in this manuscript:

AChE	Acetylcholinesterase
AChEI	Acetylcholinesterase Inhibitor
AD	Alzheimer’s Disease
ADAS-Cog	Alzheimer’s Disease Assessment Scale Cognitive
ADI	Alzheimer’s Disease International
AGs	Adapted Games
AMT	Active Music Therapy
AP2238	3-{4-[(benzylmethylamino)methyl]phenyl}-6,7-dimethoxy-2H-2-chromenone
ApoE ε4	Apolipoprotein E epsilon 4 allele
Aβ	Beta-amyloid peptide
BChE	Butyrylcholinesterase
BDI II	Beck Depression Inventory Scale-II
BPSD	Behavioral and Psychological Symptoms of Dementia
CG	Control Group
CT	Cognitive Training
D_2_/D_3_	Dopamine receptor subtypes 2 and 3
DLPFC	Dorsolateral Prefrontal Cortex
DMN	Default Mode Network
DMTs	Disease-Modifying Therapies
EEG	Electroencephalography
FAAH	Fatty Acid Amide Hydrolase
HCs	Healthy Controls
Hz	Hertz
IFNβ1a	Interferon beta-1a
IL	Interleukin
IL-10	Interleukin-10
IL-1β	Interleukin-1 beta
IL-6	Interleukin-6
LTP	Long-term potentiation
MCI	Mild Cognitive Impairment
MMSE	Mini-Mental State Examination
MRI	Magnetic Resonance Imaging
MST	Multidimensional Stimulation Group Therapy
NIBS	Non-Invasive Brain Stimulation
NMDA	N-methyl-D-aspartate
ntMST	No treated with Multidimensional Stimulation group Therapy
PC	Precuneus
PL	Placebo
PT	Physiotherapy
RCT	Randomized Controlled Trial
rTMS	Repetitive Transcranial Magnetic Stimulation
SAI	Short-latency Afferent Inhibition
tACS	Transcranial Alternating Current Stimulation
TBS	Theta Burst Stimulation
tDCS	Transcranial Direct Current Stimulation
TMS	Transcranial Magnetic Stimulation
tMST	Treated with Multidimensional Stimulation group Therapy
WG	Walking Group
WHO	World Health Organization
γ-tACS	Gamma-frequency Transcranial Alternating Current Stimulation
